# Accelerated forgetting of contextual details due to focal medio-dorsal thalamic lesion

**DOI:** 10.3389/fnbeh.2014.00320

**Published:** 2014-09-15

**Authors:** Sicong Tu, Laurie Miller, Olivier Piguet, Michael Hornberger

**Affiliations:** ^1^Neuroscience Research Australia, School of Medical Sciences, University of New South WalesSydney, NSW, Australia; ^2^Australian Research Council Centre of Excellence in Cognition and its DisordersSydney, NSW, Australia; ^3^Central Clinical School, Neuropsychology Unit, Royal Prince Alfred Hospital, University of SydneySydney, NSW, Australia; ^4^Department of Clinical Neurosciences, University of CambridgeCambridge, UK

**Keywords:** thalamus, anterograde memory, stroke, mammillothalamic tract, MRI

## Abstract

Effects of thalamic nuclei damage and related white matter tracts on memory performance are still debated. This is particularly evident for the medio-dorsal thalamus which has been less clear in predicting amnesia than anterior thalamus changes. The current study addresses this issue by assessing 7 thalamic stroke patients with consistent unilateral lesions focal to the left medio-dorsal nuclei for immediate and delayed memory performance on standard visual and verbal tests of anterograde memory, and over the long-term (>24 h) on an object-location associative memory task. Thalamic patients showed selective impairment to delayed recall, but intact recognition memory. Patients also showed accelerated forgetting of contextual details after a 24 h delay, compared to controls. Importantly, the mammillothalamic tract was intact in all patients, which suggests a role for the medio-dorsal nuclei in recall and early consolidation memory processes.

## Introduction

The thalamus is one of the major relay centers of the brain, and part of the limbic memory circuit, comprising hippocampus, fornix, mammillary bodies, mammillothalamic tract, thalamus, and cingulate cortex (Aggleton and Brown, 1999). Numerous direct and indirect projections connect mesial temporal lobe structures central for memory with the thalamus (Aggleton et al., 2011; Carlesimo et al., 2011; Preston and Eichenbaum, 2013). A well-established connection is between the hippocampus and anterior thalamic nucleus (AT), via the mammillothalamic tract (MTT). Another connection exists between the perirhinal cortex and the medio-dorsal nucleus (MD) via the inferior thalamic peduncle (Saunders et al., 2005). While amnesia is often observed in patients who have sustained a stroke involving AT or MD and the MTT (for a review see, Carlesimo et al., 2011), the unique contribution of thalamic nuclei to amnesia is still not very well understood.

With a couple of exceptions (Van der Werf et al., 2003; Perren et al., 2005), reports of memory impairment in patients with focal thalamic lesions have been mostly confined to case studies (Kishiyama et al., 2005; Edelstyn et al., 2006; Carlesimo et al., 2007; Cipolotti et al., 2008; Hampstead and Koffler, 2009). This is partly attributed to the low patient incidence, and variability in the size and location of lesions (i.e. uni- vs bi-lateral nuclei involved). Recruitment of a homogeneous sample is therefore difficult given that many patients exhibit lesions that affect multiple thalamic nuclei, namely AT and MD (Van der Werf et al., 2003; Perren et al., 2005; Cipolotti et al., 2008). Studies investigating the involvement of a single nucleus are rare and have been mostly confined to patients with damage to the AT, with a strong evidence of memory disturbance in these patients (Carlesimo et al., 2011). The impact of focal MD damage on memory is, however, virtually unexplored at the group level.

The MD is part of the “extended hippocampal system” (Aggleton and Brown, 1999). The contribution of MD damage to anterograde memory impairment following thalamic stroke is, however, less clear. A meta-analysis by Carlesimo and colleagues (2011) highlighted that only half the patients with MD lesions showed clinically diagnosed memory impairment. In contrast, all patients with lesions involving the AT were affected. Importantly, the patients with MD lesions who were amnesic showed additional damage to the MTT. Lesion to the MTT has been long known to be a strong predictor of anterograde amnesia (Vann, 2010; Carlesimo et al., 2011). While a strong relation exists between MTT damage and anterograde memory impairment (Cipolotti et al., 2008), no study has directly contrasted MTT and thalamic integrity with regard to episodic memory performance. This is of particular relevance, given the debate surrounding the contribution of the MD to anterograde memory impairment.

Characterization of the memory deficits shown in patients with focal thalamic lesions is also limited. Indeed, most studies have employed standard memory tests, which generally measure retrieval of information post encoding after a short period of time (30–60 min). In these patients, performance across memory measures indicated a prevalent deficit to delayed memory retrieval components, which has been suggested to reflect long-term anterograde memory impairment with intact short-term memory acquisition and retrieval (Carlesimo et al., 2011). Anterograde memory over the long-term (i.e., >24 h) has, however, been virtually unexplored. As such, the rate of long-term forgetting for newly acquired information has yet to be examined in thalamic stroke patients. Indeed, contrasting short vs. long-term retention would be of particular interest due to the strong structural and functional connectivity (Parker and Gaffan, 1997; Warburton et al., 2001) of the hippocampus to the thalamus, which suggests that these regions are likely to be involved in long-term memory processes. While robust evidence implicating the hippocampus in long-term memory retrieval exists from human lesion (e.g., Moscovitch et al., 2006; Bartsch et al., 2011) and functional imaging studies (Bonnici et al., 2013), the role of the thalamus in these processes is still unclear.

This study addresses these unresolved questions directly by investigating the impact of focal MD damage in a group of 7 thalamic stroke patients. More specifically, we aimed to (i) quantify MD and MTT structural integrity in these patients using voxel-based lesion mapping and diffusion tensor tractography; and (ii) relate the structural integrity to episodic memory deficits on a long-term contextual detail memory test over a 4-week period. We predicted that MD patients would show normal episodic memory deficits at short retention interval, if the MTT were intact. We also hypothesized that, even with intact MTT, MD patients would show anterograde memory impairment over the long-term in the form of accelerated forgetting due to the disruption of the limbic memory circuitry.

## Methods

### Participants

Seven individuals who had sustained a focal unilateral left thalamic stroke and self-reported memory complaints were recruited retrospectively for this study. Fifteen age- and education-matched healthy controls with no reported cognitive disturbances were also recruited. Demographics and clinical characteristics are provided in Table 1. All patients were assessed at least 3 years post-stroke. Study approval was provided by the South Eastern Sydney Local Health District Human Research Ethics Committee. All participants provided signed consent for neuropsychological assessment and neuroimaging prior to testing.

**Table 1:** **Thalamic patient and healthy control demographics, lesion localization, and performance on standardized neuropsychological assessments**.

	**Thalamic patients**							**Group average**	
	**1**	**2**	**3**	**4**	**5**	**6**	**7**	**Patients (*n* = 7)**	**Control (*n* = 15)**
								**Mean (*SD*)**	**Mean (*SD*)**
**DEMOGRAPHICS**									
Age (y.o)	64	60	71	21	59	42	40	51 (17.4)	52.2 (21.2)
Sex (M/F)	M	M	M	F	M	M	M	6 M, 1 F	7 M, 8 F
Handedness (L/R)	R	R	R	R	R	L	L	5 R, 2 L	13 R, 2 L
Education (years)	9	7	9	16	10	24	17	13.1 (6.1)	13.4 (3)
Lesion-Test Interval (years)	9	4	13	3	5	8	5	6.7 (3.5)	–
**LEFT THALAMIC NUCLEI AFFECTED**									
Anterior thalamic	–	✓	–	✓	✓	–	–	–	–
Medio-dorsal	✓	✓	✓	✓	✓	✓	✓	–	–
Ventrolateral	–	–	✓	–	–	–	✓	–	–
Normalized lesion volume (mm^3^)	90	26	166	120	206	58	298	–	–
**TESTS**									
MMSE (/30)	30	29	30	30	27	30	29	29.3 (1.1)	29.6 (0.5)
ACE-R									
Total (/100)	87	94	92	95	77	97	96	91.7 (7.1)	94.7 (5.2)
Memory (/26)	22	26	25	25	14	26	25	23.3 (4.3)	24 (2)
Doors of D&PT									
Part A (/12)	11	11	12	11	10	12	11	11.1 (0.7)	11.3 (0.8)
Part B (/12)	8	12	9	10	4	11	8	8.9 (2.6)	9.3 (2.4)
RAVLT									
T1-5 Results (/75)	36	46	31	58	22	54	49	42.3 (13)^*^	56.5 (5.9)
30 min Delay (/15)	0	8	5	13	0	11	10	6.7 (5.2)^*^	12.7 (2.1)
Recognition Correct Hits (/15)	13	13	15	14	2	15	14	12.3 (4.6)	14.1 (0.9)
RCFT									
Copy (/36)	34	28	23.5	36	35	33	28	31.1 (4.6)	31.2 (4.1)
Delayed (/36)	14	4.5	11.5	33	0.5	22.5	5	13 (11.4)	18.5 (6.8)

*MMSE, Mini-mental state examination; ACE-R, Addenbrooke's cognitive examination revised; D&PT, Doors and People Test; RAVLT, Rey auditory verbal learning test; RCFT, Rey complex figure test*.

* *Denotes significant group difference, p < 0.05. Identification of thalamic lesions was based on visual clinical ratings*.

All participants were native English speakers and were administered a battery of cognitive tests to assess overall cognitive function as well as verbal and visual memory. This assessment included: Mini-Mental State Examination (MMSE), Addenbrooke's Cognitive Examination-Revised (ACE-R), doors subtest of the Doors & People Test (D&PT), Rey Auditory Verbal Learning Test (RAVLT), and Rey Complex Figure Test (RCFT). For a brief description of cognitive tasks see Supplementary Table 1. Differences in patient and control demographics and performance on standard cognitive tests were assessed using two-tailed independent samples *t*-test in SPSS 21.0 (IBM Corp.). *P*-value < 0.05 was considered statistically significant.

Each participant also underwent a brain MRI scan. All scans were examined by a radiologist for structural abnormalities. None were reported for control participants. All thalamic stroke patients showed infarctions isolated to the thalamus. Assessment of affected thalamic nuclei was performed by LM based on clinical visual rating (Table 1). In two cases additional atrophy was present in the cerebellum (patient 2) and brain stem (patient 3).

### Long-term memory task

Episodic long-term memory was assessed using an object-based visual recognition and recall memory retrieval task. A database of 325 images depicting everyday objects on a white background was created. The word frequency of each object was calculated using the MRC Psycholinguistic Database (http://www.psych.rl.ac.uk) and images were allocated into 13 stimuli sets, each containing 25 images. Stimulus sets were matched for total word frequency, as a proxy measure of object familiarity, so that each set had a similar level of difficulty. Each participant was assigned one stimulus set to encode as target stimuli during training, with remaining sets used as novel distractors across assessments. Sets assigned as target stimuli were counterbalanced across participants.

During training, 25 target objects were presented one at a time on either the left or right hand side of a computer monitor. Participants were explicitly asked to remember the object shown as well as the side of presentation. Each target stimulus was shown for 3-s, followed by a 1-s fixation cross. This was followed immediately by a test phase. At test, stimuli consisting of the previously encoded items randomly intermixed with 25 novel items were presented centrally on the computer screen. Participants were asked to make an old/new recognition decision followed by a left/right recall decision (i.e., “Was this object shown on the left or right side of the screen?”). Responses did not have a time limit and no feedback was provided. This procedure was repeated until participants achieved at least 90% correct recognition and recall memory retrieval of the target stimuli on two consecutive training runs. All participants reached criterion within 3 training runs. For each new test run, target stimuli images were mixed with a previously unseen set of novel images, such that each set of novel stimuli was only used once throughout all assessments. Training was carried out using E-Prime 2.0 software (Psychology Software Tools, Pittsburgh, PA).

Post-training assessments were carried out via the internet by adapting the task for online assessment using WebExp (http://groups.inf.ed.ac.uk/webexp/). The test component of the task was reproduced as a java-based application accessible through any internet browser. Assessments were carried out after the following encoding delays: 1 h, 24 h, 1 week, 2 weeks, and 4 weeks. The user interface was designed to be simple and clear and each participant was provided a list of unique URL links corresponding to each online assessment. After completing each assessment a number corresponding to that session's result was generated, which participants were required to provide the examiner for data classification. The first post-training assessment was completed 1 h after the final test phase of training and under the experimenter's supervision to ensure the correct delay was followed and instructions for completing online testing was clearly understood. Scores of interest were item recognition (i.e., correct identification of whether the image was a target/novel object) and recall of contextual detail (i.e., whether the item was originally presented on the left or the right of the screen). Recognition performance was scored by the “two-high threshold model”; performance index: P_r_ (hit rate – false alarm rate); bias index: B_r_ (false alarm rate/[1-(hit rate − false alarm rate)]), values greater than 0 indicate conservative bias, values less than 0 indicate liberal bias (Snodgrass and Corwin, 1988; Soei et al., 2008; Pergola et al., 2012). Separate repeated-measures ANOVAs were carried out across all post-encoding assessments for (i) recognition (P_r_ and B_r_, independently) and (ii) recall across all participants to test for significant within and between group effects, as well as interaction effect with increasing memory retrieval delay. *Post-hoc* ANOVA contrast examined the change between each subsequent pair of time-points. Independent means comparison *t*-tests were also conducted between patients and controls for all delayed assessments. Analyses were performed using SPSS 21.0 (IBM Corp.). *P*-value < 0.05 was considered statistically significant.

### Imaging acquisition

Whole-brain T1 and diffusion weighted images were acquired for all participants using a 3T Philips MRI scanner with standard quadrature head coil (eight channels). Structural T1 scans were acquired as follows: coronal orientation, matrix 256 × 256, 180 slices, 1 mm isotropic, TE/TR = 2.5/5.4 ms, flip angle α = 8°. DTI-weighted sequences were acquired as follows: 32 gradient direction DTI sequence (repetition time/echo time/inversion time: 8400/68/90 ms; *b*-value = 1000 s/mm^2^; 55 2.5-mm horizontal slices, end resolution: 2.5 × 2.5 × 2.5 mm^3^; field of view 240 × 240 mm, 96 × 96 matrix; repeated twice) (Kwon et al., 2010). Prior to analyses, all participant scans were visually inspected for significant head movements and artifacts; none were found.

### Thalamic lesion localization

Lesions within the thalamus were manually traced on each participant's structural scan using the Harvard-Oxford Subcortical Structural Atlas in MRIcron. Individual tracings were then normalized using FSL's linear registration tool FLIRT to the MNI standard brain. Patient's normalized scans were then overlaid to provide a group profile of thalamic lesion location. Lesion volumes were calculated and correlations with patients' memory performance on standard cognitive memory tests and the long-term memory task were investigated using Pearson's correlation.

### Tractography

Probabilistic tractography was carried out to reconstruct the mammillothalamic tract using FMRIB's diffusion toolbox (FDT). DTI-weighted images were eddy-corrected by linearly registering each diffusion weighted volume to the reference T-weighted non-diffusion b0 image. All images were brain-extracted and a binary brain mask was created. Diffusion tensor models were fitted at each voxel, resulting in maps of three eigenvalues (L1, L2, L3), which allowed calculation of fractional anisotropy (FA) and mean diffusivity maps for each subject.

A high resolution DTI data set [Subject 100307; Q2 data set obtained from the MGH-UCLA Human Connectome Project (HCP) database] was used as a reference for the tractography in our participants.

Manual fiber tractography was carried out for each subject using the probabilistic cross-fiber tracking tool in FDT, PROBTRACKX. Prior to running fiber tracking, Markov Chain Monte Carlo sampling was conducted on eddy corrected DTI images to generate distributions of diffusion parameters and model crossing fibers at each voxel. Fiber tracking was then initiated with 5000 streamline samples, 0.5 mm step lengths and 0.2 curvature threshold. MTT were determined using three regions of interest (ROI): two seed regions located at the i) anterior thalamus ii) mammillary body, and a waypoint placed at the level between the mammillary body and bicommissural plane (as previously described by Kwon et al., 2010). A connectivity distribution from all voxels within the seed regions, passing through the waypoint ROI, was generated and thresholded to 30% of the maximum connectivity value to remove image noise. Thresholded fiber connectivity maps were binarized to create a mask of the MTT. Mean FA and mean diffusivity values were then calculated within each participant's fiber tract mask.

## Results

### Behavioral results

Thalamic patients and controls were well matched for age and education (*p*-values > 0.9). Thalamic patients performed in the normal range on assessments of overall cognitive function (MMSE, ACE-R: total and memory subscore) and did not differ significantly from the control group (Table 1). On standard cognitive visual memory tests, patients showed no significant differences compared to controls on the doors subtest of the D&PT and RCFT (all *p*-values > 0.1). Patient performance on the 3-min delayed recall of the RCFT varied considerably, with 5 patients performing below the 10th percentile normative score. Performance on the verbal based RAVLT, however, was significantly lower in patients than controls for immediate (T1-5), and delayed recall (*p*-values < 0.05), but not on the recognition component (*p* > 0.3).

**Figure 1:**
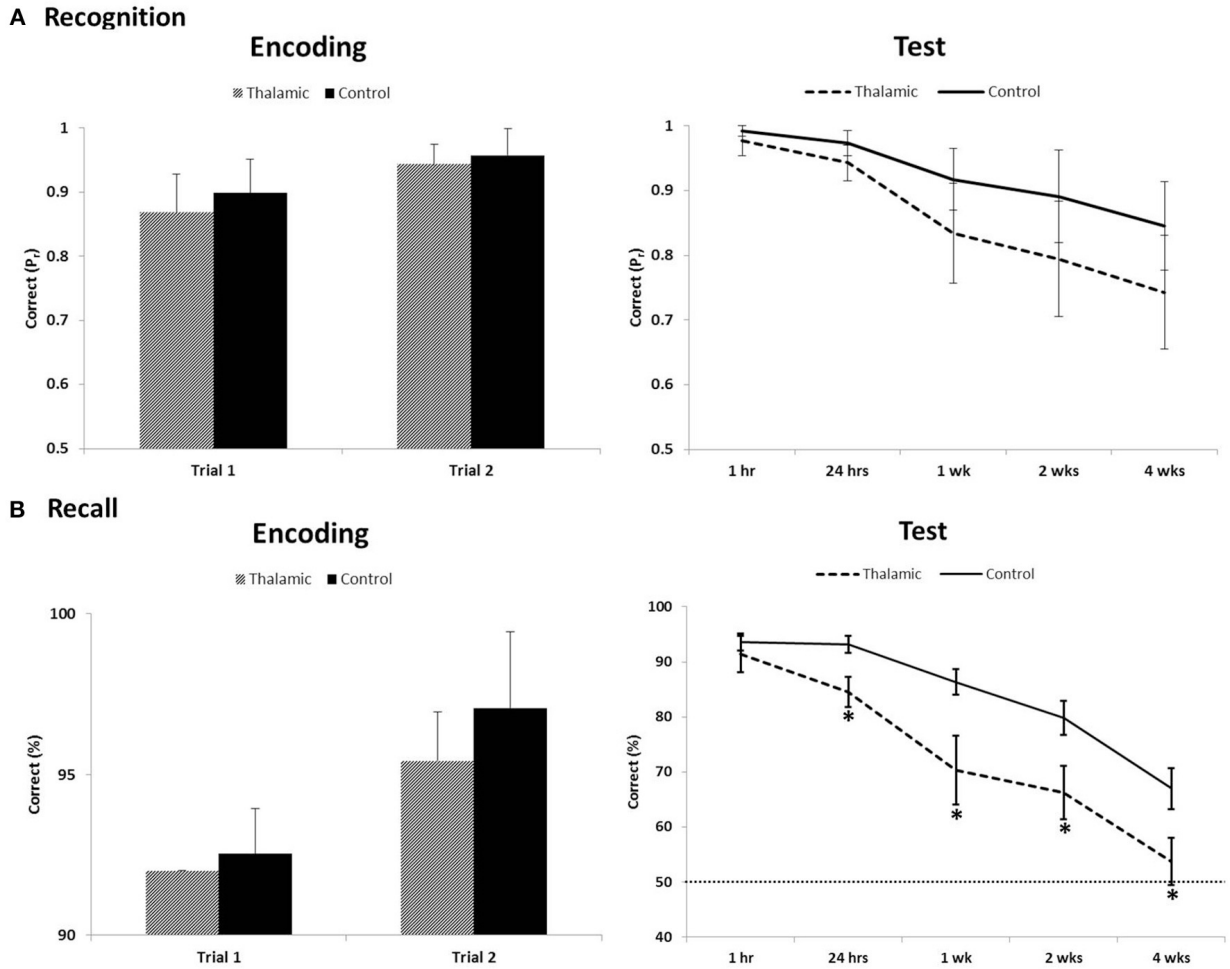
**Recognition (A) and recall (B) performance during encoding trials that met criterion (≥90%) and long-term assessment on the visual memory task between thalamic patients and controls**. Item recognition is scored as Pr (Hits − False Alarm); recall of contextual detail is scored as percent correct. Error bars indicate SE. Dotted line indicates chance performance. ^*^Indicates significant difference (*p* < 0.05).

On the long-term memory task, 12/15 controls and 5/7 patients reached the successful encoding criterion of correct item recognition and recall of contextual detail for at least 90% of stimuli, on two consecutive training trials, in the first two trials. Two patients (patient 1 and 4) and 3 controls required one additional training trial to meet criterion. Performances on successful encoding trials were compared between patients and controls. Results indicated both groups of participants learnt target stimuli to the same level for both recognition (old/new) and recall (left/right) retrieval (all *p*-values > 0.1) (Figure 1).

Repeated-measures ANOVA was carried out across all post-encoding assessments for both recognition and contextual location separately. For recognition performance index (P_r_), ANOVA indicated a significant effect of time [*F*_(2.487, 49.733)_ = 14.906, *p* < 0.001] but no group effect [*F*_(1, 20)_ = 3.4, *p* = 0.08] and no interaction [*F*_(2.487, 49.733)_ = 0.948, *p* = 0.411]. Bias index for recognition responses in patients and controls (0.26 and 0.15, respectively) showed no significant time or group effect across the 4-week delay (all *p*-values > 0.2). For contextual location, analyses revealed significant main effects of group [*F*_(1, 20)_ = 8.49, *p* = 0.009], time [*F*_(2.589, 51.778)_ = 62.71, *p* < 0.001], and a group × time interaction [*F*_(2.589, 51.778)_ = 3.797, *p* = 0.02]. *Post-hoc* contrasts, comparing change in contextual location retrieval between each subsequent assessment and the previous assessment (i.e., 1–24 h), indicated significant within-group effects across each pair of time-points (all *p*-values < 0.02). Significant between-group effects were also found across each pair of time-points (all *p*-values < 0.04) except for the final 2 week delay (2–4 weeks; *p* = 0.62). *T*-tests revealed significant group differences at all delays (*p* < 0.05), except at 1 h post-encoding (*p* = 0.38).

### Structural imaging results

**Figure 2:**
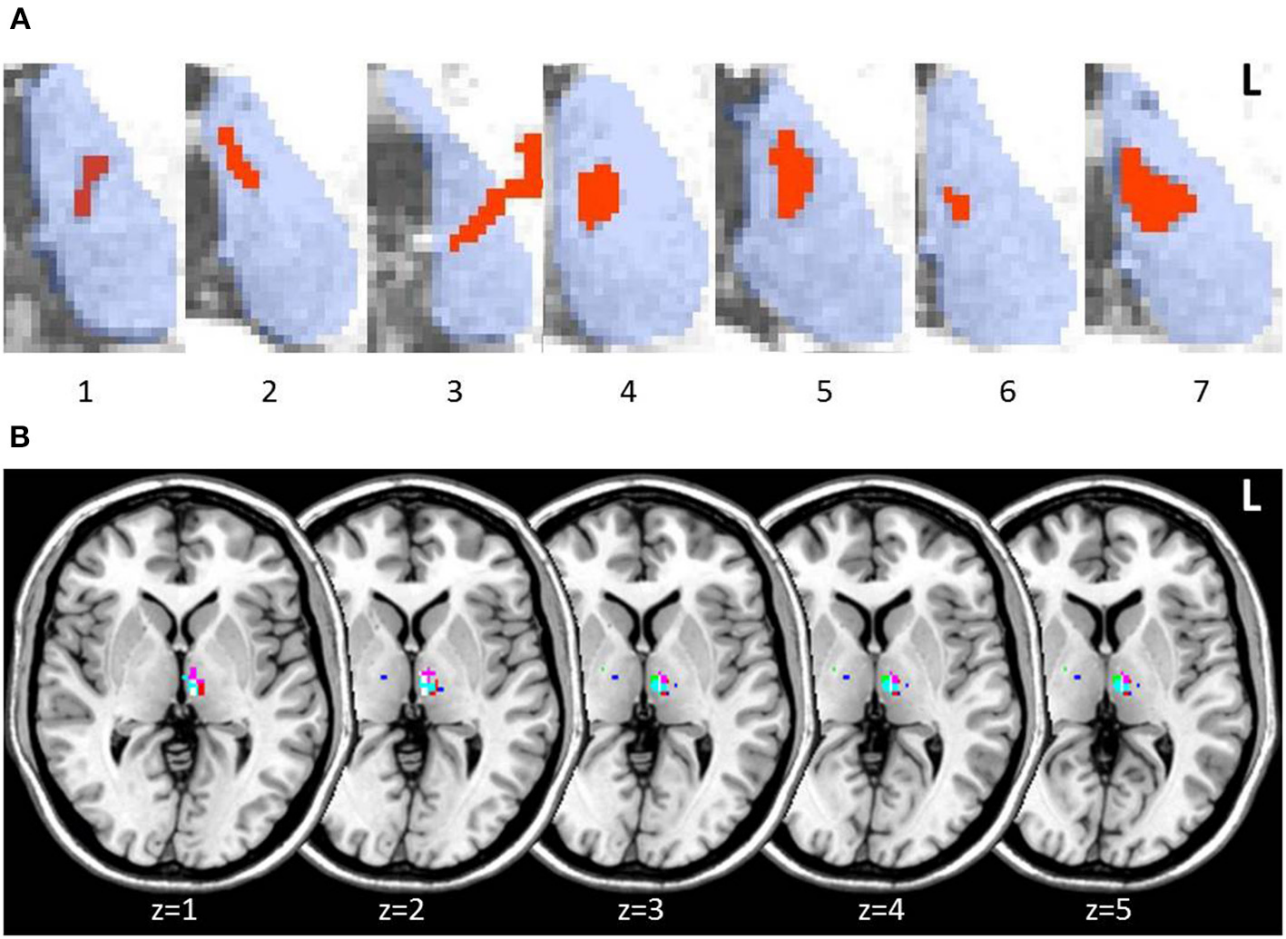
**Axial slices of thalamic patient lesions. (A)** Manual tracing of left thalamic lesion (red) on all 7 patient's structural MRI scan; outline of thalamus (blue) provided for reference. Lesions consistently involved the medio-dorsal region across patients. **(B)** Multi-slice visualization of the overlap in patient group lesions in the thalamus represented on a standard brain. Each patient's lesion is presented in a different color. Patient 3 showed minor additional lesion in the right anterior thalamus.

Lesions in thalamic patients were localized in the left thalamus, with patient 3 showing a small additional lesion in the right anterior thalamus (15 mm^3^). Manual lesion tracing from patients' structural scans showed lesions affecting the left MD in all patients (Figure 2A). When we created a group lesion representation, after normalizing patient scans onto a standard brain, left MD was the only region to show overlap on the group lesion map (Figure 2B).

The MD lesion in 3 patients (2, 4, and 5) partially extended into the AT. Overall, total thalamic lesion volumes did not correlate with memory performance on standard memory tests or the long-term memory task (all *p*-values > 0.1). Patient 5, however, who performed the lowest across all standardized assessments of memory, was the individual with the largest lesion involving AT (though not the largest total thalamic lesion). We performed the same behavioral analyses between patients and controls on patients with and without AT involvement. No significant differences in performance were detected on any of the standard cognitive tests or across delayed assessments on the long-term memory task (all *p*-values > 0.1; Supplementary Figure 1).

### Probabilistic tractography results

The MTT was individually reconstructed in both hemispheres in patients and controls (Supplementary Figure 2). The tract was first reconstructed using HCP data to obtain the anatomical pathway and a guide for reconstruction in our data. The tract runs posterior to the column of the fornix and lateral from the mammillary bodies to the anterior thalamus. Group FA, mean diffusivity, and tract volume of the MTT were extracted from both hemispheres (Table 2). Diffusion measures (FA, mean diffusivity) of the MTT were consistent across patients, including those with additional lesion involvement of the AT. Independent samples *t*-test revealed no significant differences in FA, mean diffusivity or tract volume between thalamic patients and the control group (all *p*-values > 0.2). No significant correlations were present between memory retention (i.e., change in memory performance across the 4-week delay) and MTT integrity as measured by FA or mean diffusivity.

**Table 2:** **Mean FA, mean diffusivity and tract volume of the mammillothalamic tract in thalamic patients and controls**.

	**Thalamic patients**	**Control**	**Th vs. Con**
	**Mean**	**Mean**	***P*-value**
**FA**			
Left MTT	0.38 (0.06)	0.36 (0.04)	0.20
Right MTT	0.36 (0.07)	0.35 (0.04)	0.52
**MEAN DIFFUSIVITY**			
Left MTT	0.8 (0.1)	0.79 (0.09)	0.79
Right MTT	0.78 (0.0)	0.8 (0.09)	0.58
**TRACT VOLUME**			
Left MTT	135 (20)	135 (21)	0.94
Right MTT	129 (26)	127 (15)	0.83

*Mean and SD reported. FA, fractional anisotropy; mean diffusivity, × 10^−3^ (mm^2^/s)*.

## Discussion

Investigations of episodic memory integrity following focal thalamic stroke reveal that focal left MD thalamic lesion impair long-term (>24 h), but not short-term recognition memory even in the absence of MTT involvement. This finding was reflected by accelerated forgetting of newly acquired contextual location after 24 h, compared to healthy controls.

The general cognitive profile of our thalamic stroke patients on standard neuropsychological tests of memory is consistent with previous reports. Specifically, lesions to the left thalamus consistently impaired memory for verbal more than visual stimuli (Shim et al., 2008; Nishio et al., 2011). Patients also showed marked impairment to delayed (<1 h) memory recall on tasks with challenging encoding requirements (RCFT and RAVLT). When the task requirements were easier (ACE-R and the long-term memory task) performance was intact (Carlesimo et al., 2011). Notably, patients performed to a similar level as controls on the memory component of the ACE-R which requires participants to recall a name and address after a 15 min delay. In contrast, on the RAVLT, when the delay in recall is extended to 30 min, on a 15 item unstructured list, patients performed noticeably worse than controls. The RAVLT is a more cognitively demanding task and impaired patient performance, compared to controls, was also evident in immediate recall trials. This essentially reflects a difference in the level of encoding between patients and controls. Nevertheless, intact delayed recall on the ACE-R suggests the initial encoding, and short-term retention, of simple stimuli to episodic memory are intact, a finding also observed in the long-term visual memory task.

The long-term anterograde memory task we employed assessed participant recognition of visual objects and recall of their associated screen locations (left vs. right side of the screen) over a 4-week period. Patients were able to match controls in performance during encoding and also after a 1-h delay on both recognition and contextual detail retrieval. This was consistent with their memory profile derived from cognitive testing, that acquisition and short-term memory retention for simple stimuli is intact. When the delay is extended beyond 24 h, patients show a rapid rate of forgetting contextual detail at 24 h post-encoding that persists throughout follow-up testing, performing near chance level by the end of the 4-week period. In contrast, controls correctly remembered ~70% of the locations of target stimuli after 4 weeks. Performance on the recognition component was intact, suggesting that there may be dissociation in long-term retrieval of recall and recognition aspects of memory. The observed dissociation may, however, also be due to an inherent difference in difficulty between task components, despite similar levels of encoding. This pattern of memory impairment coupled with focal lesion to the MD of the thalamus is, however, not consistent with the proposed role of thalamic nuclei in the limbic memory circuit (Aggleton and Brown, 1999).

In Aggleton and Brown's (1999) influential model the “extended hippocampal system,” recall of contextual detail such as specific spatial and/or temporal association is more associated with medial temporal lobe and AT neural connections whereas recognition of an object or item is associated with perirhinal cortex and the MD region. Consistent with the hypothesis, case-study reports of patients with MTT and AT infarctions have reported severe impaired recall, but intact recognition on verbal and visual test modalities (Edelstyn et al., 2002, 2006; Carlesimo et al., 2007). In contrast, focal MD damage resulting in intact recognition but impaired recall has yet to be directly shown. Rather patient reports indicate impairment across both recall and recognition, to varying degrees, following MD damage (Kishiyama et al., 2005; Cipolotti et al., 2008; Soei et al., 2008; Pergola et al., 2012). Recent reviews of the memory literature, have also failed to find strong evidence for such a dissociation (Aggleton et al., 2011; Carlesimo et al., 2011). To our knowledge, two previous studies have also shown impaired recall using a contextual memory task, although with immediate recall, when the MD is damaged (Soei et al., 2008; Pergola et al., 2012). Alternatively, functional neuroimaging (de Rover et al., 2008; Pergola et al., 2013) has shown that the MD is more involved in recall than recognition. These lesion and fMRI findings dovetail with our current findings, on a group level, showing impaired recall of contextual detail but relatively intact object recognition memory performance. More importantly, lesion volume in patients was not correlated with memory performance in our study, although extension into the AT region, in one patient, resulted in greater memory impairment across cognitive assessments. Therefore, the current findings suggest that the memory functions attributed to sub-thalamic nuclei (AT: recall processes; MD: recognition processes) may be oversimplified. A graded involvement of multiple thalamic nuclei in the recall and recognition dichotomy (Aggleton et al., 2011; Pergola et al., 2012) is more consistent with the current findings.

In addition, we were able to directly rule out MTT involvement for the memory impairment observed in our cohort of patients. Indeed, diffusion measures and tract volume were similar in patients and controls, and showed consistency within groups across hemispheres. Integrity of the MTT in the three patients with additional AT involvement also did not differ from remaining patients. To our knowledge, this is the first study to quantify the integrity of the mammillothalamic tract consistently in thalamic stroke patients on a group level. Diffusion imaging of the MTT provided an objective method of characterizing the extent of lesions in thalamic stroke and their disruption to the limbic memory circuit. In particular, it allowed us to dissociate the extent of thalamic nuclei and MTT contributions to memory impairment.

Importantly, the MD has strong connections to the dorsal lateral prefrontal cortex (DLPFC) (Aggleton et al., 2011). The specific role of the DLPFC in episodic memory processes is not fully established; however, recent functional neuroimaging evidence has shown that the DLPFC is particularly involved in contextual encoding, especially for the association of between-items compared to associations of within-item features (Blumenfeld et al., 2011). Further, Pergola and colleagues (2013) have shown that the DLPFC and MD are both activated during associative memory retrieval. The contribution of DLPFC and MD connectivity to long-term episodic memory, however, remains unclear. One potential avenue to investigate this in the future would be to contrast thalamic lesion with DLPFC lesion patients directly to elucidate the contributions of each region.

The observed change in retrieval of contextual detail between our thalamic patients and controls in the first week, despite reaching the same level of encoding, behaviorally resembles the accelerated long-term forgetting (ALF) phenomenon identified in some patients with temporal lobe epilepsy (for a review see, Fitzgerald et al., 2013). Notably, some patients show rapid forgetting from as early as 24 h despite normal learning and initial retention, and intact hippocampus (Mayes et al., 2003; Muhlert et al., 2010). ALF is, therefore, believed to represent a consolidation deficit rather than impaired acquisition (Hoefeijzers et al., 2013). No prior studies have implicated the MD in ALF, although the AT has been shown to be a crucial structure for seizure propagation in temporal lobe epilepsy (Mueller et al., 2010). Interestingly, Vilberg and Davachi (2013) have recently demonstrated, using a verbal word and object/scene memory task, increased connectivity between the perirhinal cortex and hippocampus for selective consolidation of object based memory. Our findings also suggest that the MD plays a significant role in long-term memory processes, and might be relevant for early stages of consolidation. Similar to the role multiple thalamic nuclei play in declarative memory (Aggleton et al., 2011), they may also serve roles in memory as a function of time in the HC-cortical consolidation process. The function of the thalamus as a relay structure in the consolidation process, however, has yet to be included in theoretical models, which remain focused on the hippocampus (Squire and Wixted, 2011; Winocur and Moscovitch, 2011). This may be due to a number of factors, (i) when the MTT is not involved, patients with focal damage to the thalami show a more varied and milder anterograde memory impairment (Carlesimo et al., 2011) that is not detected across a number of standard cognitive tests and not investigated over the long-term, (ii) anatomical connections leading to thalamic nuclei from medial temporal lobe structures, and from thalamic nuclei to specific cortical regions remains unclear in humans, and (iii) absence of functional imaging studies implicating the thalamus in consolidation processes.

Despite these promising findings, some methodological limitations warrant attention. In particular, despite very careful lesion mapping, we cannot exclude further thalamic damage on a cellular level which could have affected our results. Clearly, intra-thalamic connectivity is complex and thus our results may have been influenced by changes which were not picked up macroscopically. The MTT tractography analysis was conducted according to published protocols (Kwon et al., 2010), however the tract is very small and therefore voxel size of DTI acquisition and partial volume effects might have affected our findings. We addressed accuracy issues by first reconstructing the tract in a high resolution Human Connectome Project data set to visually assess overall shape and anatomical location of the tract in our data. Tract volumes of the MTT were bigger than those reported in previous studies (Cipolotti et al., 2008; Kwon et al., 2010), likely due to a larger voxel size acquisition of our DTI data. Obtained FA and mean diffusivity values were, however, consistent across hemispheres and comparable to those reported by Kwon and colleagues (2010), who reliably reconstructed the tract in 25 healthy young controls.

In conclusion, unilateral lesion focal to the left medio-dorsal nuclei of the thalamus, in the absence of damage to the mammillothalamic tract, impairs anterograde memory recall. The findings support the notion that the medio-dorsal nuclei play a role in long-term delayed retrieval of recall type memory processes.

### Conflict of interest statement

The authors declare that the research was conducted in the absence of any commercial or financial relationships that could be construed as a potential conflict of interest.

## References

[B1] Aggleton J. P. Brown M. W. (1999). Episodic memory, amnesia, and the hippocampal-anterior thalamic axis. Behav. Brain Sci. 22, 425–444 discussion: 444–489. 10.1017/S0140525X99002034 11301518

[B2] Aggleton J. P. Dumont J. R. Warburton E. C. (2011). Unraveling the contributions of the diencephalon to recognition memory: a review. Learn. Mem. 18, 384–400 10.1101/lm.1884611 21597044 PMC3101772

[B3] Bartsch T. Dohring J. Rohr A. Jansen O. Deuschl G. (2011). CA1 neurons in the human hippocampus are critical for autobiographical memory, mental time travel, and autonoetic consciousness. Proc. Natl. Acad. Sci. U.S.A. 108, 17562–17567 10.1073/pnas.1110266108 21987814 PMC3198338

[B4] Blumenfeld R. S. Parks C. M. Yonelinas A. P. Ranganath C. (2011). Putting the pieces together: the role of dorsolateral prefrontal cortex in relational memory encoding. J. Cogn. Neurosci. 23, 257–265 10.1162/jocn.2010.21459 20146616 PMC3970078

[B5] Bonnici H. M. Chadwick M. J. Maguire E. A. (2013). Representations of recent and remote autobiographical memories in hippocampal subfields. Hippocampus 23, 849–854 10.1002/hipo.22155 23749406 PMC4281962

[B6] Carlesimo G. A. Lombardi M. G. Caltagirone C. (2011). Vascular thalamic amnesia: a reappraisal. Neuropsychologia 49, 777–789 10.1016/j.neuropsychologia.2011.01.026 21255590

[B7] Carlesimo G. A. Serra L. Fadda L. Cherubini A. Bozzali M. Caltagirone C. (2007). Bilateral damage to the mammillo-thalamic tract impairs recollection but not familiarity in the recognition process: a single case investigation. Neuropsychologia 45, 2467–2479 10.1016/j.neuropsychologia.2007.03.025 17512561

[B8] Cipolotti L. Husain M. Crinion J. Bird C. M. Khan S. S. Losseff N. (2008). The role of the thalamus in amnesia: a tractography, high-resolution MRI and neuropsychological study. Neuropsychologia 46, 2745–2758 10.1016/j.neuropsychologia.2008.05.009 18597798

[B9] de Rover M. Petersson K. M. van der Werf S. P. Cools A. R. Berger H. J. Fernandez G. (2008). Neural correlates of strategic memory retrieval: differentiating between spatial-associative and temporal-associative strategies. Hum. Brain Mapp. 29, 1068–1079 10.1002/hbm.20445 17948888 PMC6870844

[B10] Edelstyn N. M. Ellis S. J. Jenkinson P. Sawyer A. (2002). Contribution of the left dorsomedial thalamus to recognition memory: a neuropsychological case study. Neurocase 8, 442–452 10.1076/neur.8.5.442.16180 12529453

[B11] Edelstyn N. M. Hunter B. Ellis S. J. (2006). Bilateral dorsolateral thalamic lesions disrupts conscious recollection. Neuropsychologia 44, 931–938 10.1016/j.neuropsychologia.2005.08.012 16253293

[B12] Fitzgerald Z. Mohamed A. Ricci M. Thayer Z. Miller L. (2013). Accelerated long-term forgetting: a newly identified memory impairment in epilepsy. J. Clin. Neurosci. 20, 1486–1491 10.1016/j.jocn.2013.04.037 24076316

[B13] Hampstead B. M. Koffler S. P. (2009). Thalamic contributions to anterograde, retrograde, and implicit memory: a case study. Clin. Neuropsychol. 23, 1232–1249 10.1080/13854040902936679 19548181

[B14] Hoefeijzers S. Dewar M. Della Sala S. Zeman A. Butler C. (2013). Accelerated long-term forgetting in transient epileptic amnesia: an acquisition or consolidation deficit? Neuropsychologia 51, 1549–1555 10.1016/j.neuropsychologia.2013.04.017 23651707

[B15] Kishiyama M. M. Yonelinas A. P. Kroll N. E. Lazzara M. M. Nolan E. C. Jones E. G. (2005). Bilateral thalamic lesions affect recollection- and familiarity-based recognition memory judgments. Cortex 41, 778–788 10.1016/S0010-9452(08)70296-X 16353367

[B16] Kwon H. G. Hong J. H. Jang S. H. (2010). Mammillothalamic tract in human brain: diffusion tensor tractography study. Neurosci. Lett. 481, 51–53 10.1016/j.neulet.2010.06.052 20599587

[B17] Mayes A. R. Isaac C. L. Holdstock J. S. Cariga P. Gummer A. Roberts N. (2003). Long-term amnesia: a review and detailed illustrative case study. Cortex 39, 567–603 10.1016/S0010-9452(08)70855-4 14584544

[B18] Moscovitch M. Nadel L. Winocur G. Gilboa A. Rosenbaum R. S. (2006). The cognitive neuroscience of remote episodic, semantic and spatial memory. Curr. Opin. Neurobiol. 16, 179–190 10.1016/j.conb.2006.03.013 16564688

[B19] Mueller S. G. Laxer K. D. Barakos J. Cheong I. Finlay D. Garcia P. (2010). Involvement of the thalamocortical network in TLE with and without mesiotemporal sclerosis. Epilepsia 51, 1436–1445 10.1111/j.1528-1167.2009.02413.x 20002143 PMC2888933

[B20] Muhlert N. Milton F. Butler C. R. Kapur N. Zeman A. Z. (2010). Accelerated forgetting of real-life events in Transient Epileptic Amnesia. Neuropsychologia 48, 3235–3244 10.1016/j.neuropsychologia.2010.07.001 20620156

[B21] Nishio Y. Hashimoto M. Ishii K. Mori E. (2011). Neuroanatomy of a neurobehavioral disturbance in the left anterior thalamic infarction. J. Neurol. Neurosurg. Psychiatry 82, 1195–1200 10.1136/jnnp.2010.236463 21515557 PMC3188785

[B22] Parker A. Gaffan D. (1997). Mamillary body lesions in monkeys impair object-in-place memory: functional unity of the fornix-mamillary system. J. Cogn. Neurosci. 9, 512–521 10.1162/jocn.1997.9.4.512 23968214

[B23] Pergola G. Gunturkun O. Koch B. Schwarz M. Daum I. Suchan B. (2012). Recall deficits in stroke patients with thalamic lesions covary with damage to the parvocellular mediodorsal nucleus of the thalamus. Neuropsychologia 50, 2477–2491 10.1016/j.neuropsychologia.2012.06.019 22750446

[B24] Pergola G. Ranft A. Mathias K. Suchan B. (2013). The role of the thalamic nuclei in recognition memory accompanied by recall during encoding and retrieval: an fMRI study. Neuroimage 74, 195–208 10.1016/j.neuroimage.2013.02.017 23435209

[B25] Perren F. Clarke S. Bogousslavsky J. (2005). The syndrome of combined polar and paramedian thalamic infarction. Arch. Neurol. 62, 1212–1216 10.1001/archneur.62.8.1212 16087760

[B26] Preston A. R. Eichenbaum H. (2013). Interplay of hippocampus and prefrontal cortex in memory. Curr. Biol. 23, R764–R773 10.1016/j.cub.2013.05.041 24028960 PMC3789138

[B27] Saunders R. C. Mishkin M. Aggleton J. P. (2005). Projections from the entorhinal cortex, perirhinal cortex, presubiculum, and parasubiculum to the medial thalamus in macaque monkeys: identifying different pathways using disconnection techniques. Exp. Brain Res. 167, 1–16 10.1007/s00221-005-2361-3 16143859

[B28] Shim Y. S. Kim J. S. Shon Y. M. Chung Y. A. Ahn K. J. Yang D. W. (2008). A serial study of regional cerebral blood flow deficits in patients with left anterior thalamic infarction: anatomical and neuropsychological correlates. J. Neurol. Sci. 266, 84–91 10.1016/j.jns.2007.09.016 18031760

[B29] Snodgrass J. G. Corwin J. (1988). Pragmatics of measuring recognition memory: applications to dementia and amnesia. J. Exp. Psychol. Gen. 117, 34–50 10.1037/0096-3445.117.1.34 2966230

[B30] Soei E. Koch B. Schwarz M. Daum I. (2008). Involvement of the human thalamus in relational and non-relational memory. Eur. J. Neurosci. 28, 2533–2541 10.1111/j.1460-9568.2008.06536.x 19032591

[B31] Squire L. R. Wixted J. T. (2011). The cognitive neuroscience of human memory since H.M. Annu. Rev. Neurosci. 34, 259–288 10.1146/annurev-neuro-061010-113720 21456960 PMC3192650

[B32] Van der Werf Y. D. Scheltens P. Lindeboom J. Witter M. P. Uylings H. B. Jolles J. (2003). Deficits of memory, executive functioning and attention following infarction in the thalamus; a study of 22 cases with localised lesions. Neuropsychologia 41, 1330–1344 10.1016/S0028-3932(03)00059-9 12757906

[B33] Vann S. D. (2010). Re-evaluating the role of the mammillary bodies in memory. Neuropsychologia 48, 2316–2327 10.1016/j.neuropsychologia.2009.10.019 19879886

[B34] Vilberg K. L. Davachi L. (2013). Perirhinal-hippocampal connectivity during reactivation is a marker for object-based memory consolidation. Neuron 79, 1232–1242 10.1016/j.neuron.2013.07.013 23993700 PMC3837480

[B35] Warburton E. C. Baird A. Morgan A. Muir J. L. Aggleton J. P. (2001). The conjoint importance of the hippocampus and anterior thalamic nuclei for allocentric spatial learning: evidence from a disconnection study in the rat. J. Neurosci. 21, 7323–7330 11549742 10.1523/JNEUROSCI.21-18-07323.2001 PMC6762976

[B36] Winocur G. Moscovitch M. (2011). Memory transformation and systems consolidation. J. Int. Neuropsychol. Soc. 17, 766–780 10.1017/S1355617711000683 21729403

