# Supplementary material for: Accelerated forgetting of contextual details due to focal medio-dorsal thalamic lesion
Source: Front Behav Neurosci. 2014 Sep 15;8:320. doi: 10.3389/fnbeh.2014.00320 (PMC4163931; doi:10.3389/fnbeh.2014.00320)
Supplement: Supplementary file 1 [file DataSheet1.DOCX]

**Supplementary Table 1.** Description of neuropsychological tasks administered.

| **Neuropsychological Test** | **Task Components** |
| --- | --- |
| Addenbrooke Cognitive Exam –Revised (ACE-R)  (Mioshi et al., 2006) | The ACE-R is a general screening measure of cognition scored out of 100 and contains sub-components assessing: attention and orientation, memory, verbal fluency, language, and visuospatial skills. The memory subtest score comprises (1) recall after brief distracton of a three-item list, (2) recall of a seven-item name and address on the third learning trial, (3) delayed recall and recognition of the name and address, (4) recall of the names of 4 specified current and previous politicians. |
| Doors subtest of the Doors & People Test (D&PT)  (Baddeley, Emslie, & Nimmo-Smith, 1994) | This subtest examines visual recognition memory. It is comprised of two sections (A/B). In each section participants are first shown pictures of 12 different doors, then required to pick them out one at a time from arrays of 4 pictures (target and 3 distractors). In section B, the targets and distracters are more closely matched than in section A, and thus, more difficult. |
| Rey Auditory Verbal Learning Test (RAVLT)  (Schmidt, 1996) | RAVLT is a measure of episodic memory recall for verbal information.  ***A1-5***: a list of 15 words is read aloud over five consecutive trials, each followed by a free recall test  ***B1***: a second ‘interference’ list of 15 words is read aloud followed by a free recall test  ***A6***: participants are required to recall words from the first list again  ***30 min Delayed recall***: 30min after A6, participants are asked to recall words from the first list  ***Recognition***: after the delayed recall test, participants perform a recognition test containing all items from the first and interference lists in addition to 20 new words. They are asked to say yes or no as to whether each word occurred on the first list. |
| Rey-Osterrieth Complex Figure Test (RCFT)  (Meyers & Meyers, 1995) | RCFT is a measure of episodic memory recall for visual information.  ***Copy***: participants are asked to copy a complex figure as accurately as possible  ***Delayed***: 3 minutes after copying, participants are instructed to reproduce the figure from memory |

References

Baddeley, A. D., Emslie, H., & Nimmo-Smith, I. (1994). The Doors and People Test: a test of visual and verbal recall and recognition. Bury St. Edmonds: Thames Valley Test Company.

Meyers, J., & Meyers, K. (1995). *The Meyers Scoring System for the Rey Complex Figure and the Recognition Trial: Professional Manual*. Odessa, FL: Psychological Assessment Resources.

Mioshi, E., Dawson, K., Mitchell, J., Arnold, R., & Hodges, J. R. (2006). The Addenbrooke's Cognitive Examination Revised (ACE-R): a brief cognitive test battery for dementia screening. *Int J Geriatr Psychiatry, 21*(11), 1078-1085.

Schmidt, M. (1996). *Rey Auditory and Verbal Learning Test: A Handbook*. Los Angeles: Western Psychological Services.

**
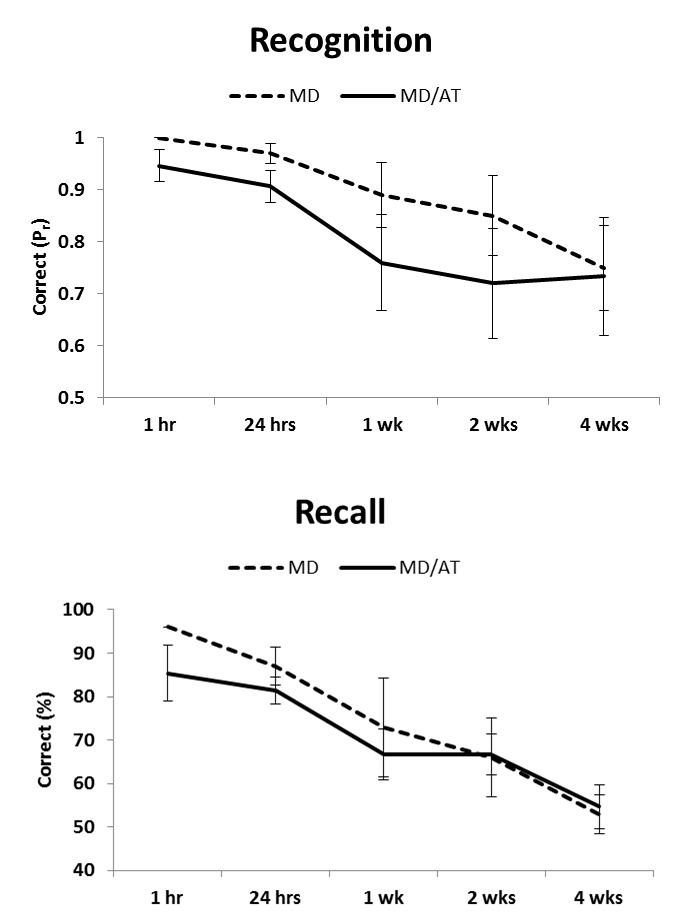
**

**Supplementary Figure 1.** Three patients with MD lesion extending into AT (MD/AT) do not perform significantly different to those with lesion only to the MD in item recognition and recall of contextual detail on the long-term memory task.


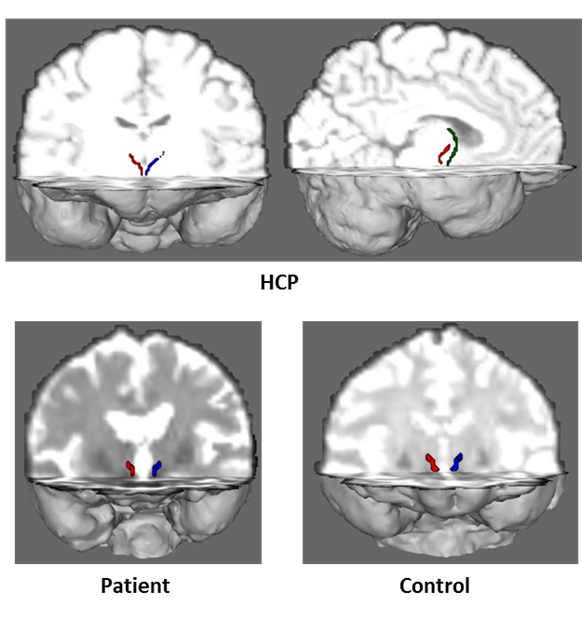


**Supplementary Figure 2.** 3D rendering of reconstructed mammillothalamic tract in both hemispheres (red: right; blue: left) using a data set from the Human Connectome Project (HCP; column of the fornix in green) and representative mammillothalamic tracts in a thalamic patient and control.
